# Comprehensive therapy for human H5N1

**DOI:** 10.1186/1753-6561-5-S1-P10

**Published:** 2011-01-10

**Authors:** Jin Takasaki, Shinyu Izumi, Nguyen Dang Tuan, Dao Xuan Co, Dang Hung Minh, Ngo Quy Chau, Nguyen Gia Binh, Vu Thi Van, Toshie Manabe, Pham Thi Phuong Thuy, Tran Thuy Hanh, Koichiro Kudo

**Affiliations:** 1 Disease Control and Prevention Center (DCC)/ Department of Pulmonary Medicine, National Center for Global Health and Medicine (NCGM), Tokyo, 162-8655 Japan; 2 Back Mai Hospital, Hanoi, 100000 Vietnam

## Introduction

The mortality of human H5N1 (pneumonia) is very high. The cumulative number of confirmed human cases of avian influenza A(H5N1) reported is 504, and 59.3% of them have been dead (situation on 12, August 2010). Early intervention could improve the prognosis of this disease.

## Methods

In order to initiate appropriate treatment earlier, we perform a three step strategy named “Comprehensive Therapy for human A (H5N1): CT-H5N1” in northern Vietnam since 2008. Upon step 1, residents are educated to visit healthcare facilities sooner when he/she gets sick after close contact with sick/dead poultry; on Step 2, medical staffs in provincial hospitals make a diagnosis using a rapid diagnostic test for A(H5N1) that our colleagues developed. If positive, the patient is initiated antiviral treatments immediately and is referred to Central Hospital (BMH), if he/she is in severe condition; and on Step 3, the patient in critical condition is given advanced and intensive treatments including blood purification therapy.

## Results

Since December, 2008, we have been able to enroll 3 patients into our collaborative clinical research. All of them successfully survive.

## Conclusion

CT-H5N1 would be effective; however enrollment of more patients is desirable.

